# ASO Author Reflections: Risk Estimation vs. Real-World Practice: SLNB Decision-Making in Thin Melanoma

**DOI:** 10.1245/s10434-026-19199-8

**Published:** 2026-02-10

**Authors:** Mackenzie M. Mayhew, Russell G. Witt

**Affiliations:** Department of Surgery, University of Virginia, Charlottesville, VA USA

## Past

Sentinel lymph node biopsy (SLNB) is a cornerstone of melanoma staging; yet its use in thin melanoma remains an area of clinical uncertainty where both overuse and underuse are concerns. Current National Comprehensive Cancer Network guidelines rely primarily on Breslow depth and ulceration to guide SLNB recommendations, while acknowledging that additional pathologic and patient-specific factors influence nodal metastasis risk.^1^ To support more individualized risk assessment, validated prediction tools such as the Melanoma Institute of Australia (MIA) sentinel node metastasis nomogram were developed to estimate nodal metastasis risk using multiple tumor- and patient-level variables.^2,3^

## Present

In this national analysis of patients with thin melanoma (<1.0 mm Breslow thickness), we observed significant discordance between predicted nodal metastasis risk and SLNB utilization both before and after dissemination of the MIA nomogram. Approximately one-third of patients classified as high risk (>10% predicted risk) did not undergo SLNB, while 14% of patients classified as low risk (<5% predicted risk) underwent the procedure.^4^ This discordance reflects both potential underutilization and overutilization of SLNB. Sentinel lymph node biopsy utilization patterns changed minimally following publication of the MIA model, suggesting limited real-world adoption.

Instead, SLNB use remained more strongly associated with traditional tumor-specific factors, such as Breslow thickness, ulceration, and mitotic rate, than with integrated risk estimates incorporating patient age and other variables. This discordance may reflect patient preference, clinician reliance on alternative risk assessment strategies, or differences in how risk factors are weighted in real-world decision-making.

## Future

Future efforts should prioritize implementation of validated nodal risk calculators within routine clinical workflows rather than further refinement of prediction models alone. Risk calculators, such as the MIA nomogram, are widely available at no cost and could be integrated into electronic health records and shared decision-making discussions to better align SLNB utilization with individualized metastatic risk.
